# Climate-Induced Northerly Expansion of Siberian Silkmoth Range

**DOI:** 10.3390/f8080301

**Published:** 2017-08-16

**Authors:** Viacheslav I. Kharuk, Sergei T. Im, Kenneth J. Ranson, Mikhail N. Yagunov

**Affiliations:** 1 V.N. Sukachev Institute of Forest SB RAS, Federal Research Center Krasnoyarsk Science Center SB RAS Academgorodok 50/28, 660036 Krasnoyarsk, Russia; 2 Siberian Federal University, Institute of Space and Information Technology, pr. Kirenskogo 26a, 660074 Krasnoyarsk, Russia; 3 Siberian Federal University, Institute of Ecology and Geography, pr. Svobodny 79, 660041 Krasnoyarsk, Russia; 4 Reshetnev Siberian State University of Science and Technology, Institute of Space Research and High Technologies, pr. Krasnoyarskiy Rabochii 31, 660014 Krasnoyarsk, Russia; 5 NASA s Goddard Space Flight Center, Greenbelt, MD 20771, USA; 6 Russian Center of Forest Protection, 660036 Krasnoyarsk, Russia

**Keywords:** Siberian silkmoth, climate change, phyllophages, pest outbreaks, biotic impact on forests, Siberian taiga, climate impact on insects

## Abstract

Siberian silkmoth (*Dendrolimus sibiricus* Tschetv.) is a dangerous pest that has affected nearly 2.5 × 10^6^ ha of “dark taiga” stands (composed of *Abies sibirica*, *Pinus sibirica* and *Picea obovata*) within the latitude range of 52°–59° N. Here we describe a current silkmoth outbreak that is occurring about half degree northward of its formerly documented outbreak range. This outbreak has covered an area of about 800 thousand ha with mortality of conifer stands within an area of about 300 thousand ha. The primary outbreak originated in the year 2014 within stands located on gentle relatively dry southwest slopes at elevations up to 200 m above sea level (a.s.l.) Then the outbreak spread to the mesic areas including northern slopes and the low-elevation forest belts along the Yenisei ridge. Within the outbreak area, the northern Siberian silkmoth population has reduced generation length from two to one year. Our study showed that the outbreak was promoted by droughts in prior years, an increase of the sum of daily temperatures (*t* > +10 °C), and a decrease in ground cover moisture. Within the outbreak area, secondary pests were also active, including the aggressive *Polygraphus proximus* bark borer beetle. The outbreak considered here is part of the wide-spread (panzonal) Siberian silkmoth outbreak that originated during 2014–2015 with a range of up to 1000 km in southern Siberia. Our work concludes that observed climate warming opens opportunities for Siberian silkmoth migration into historically outbreak free northern “dark taiga” stands.

## 1. Introduction

**Figure 1.**
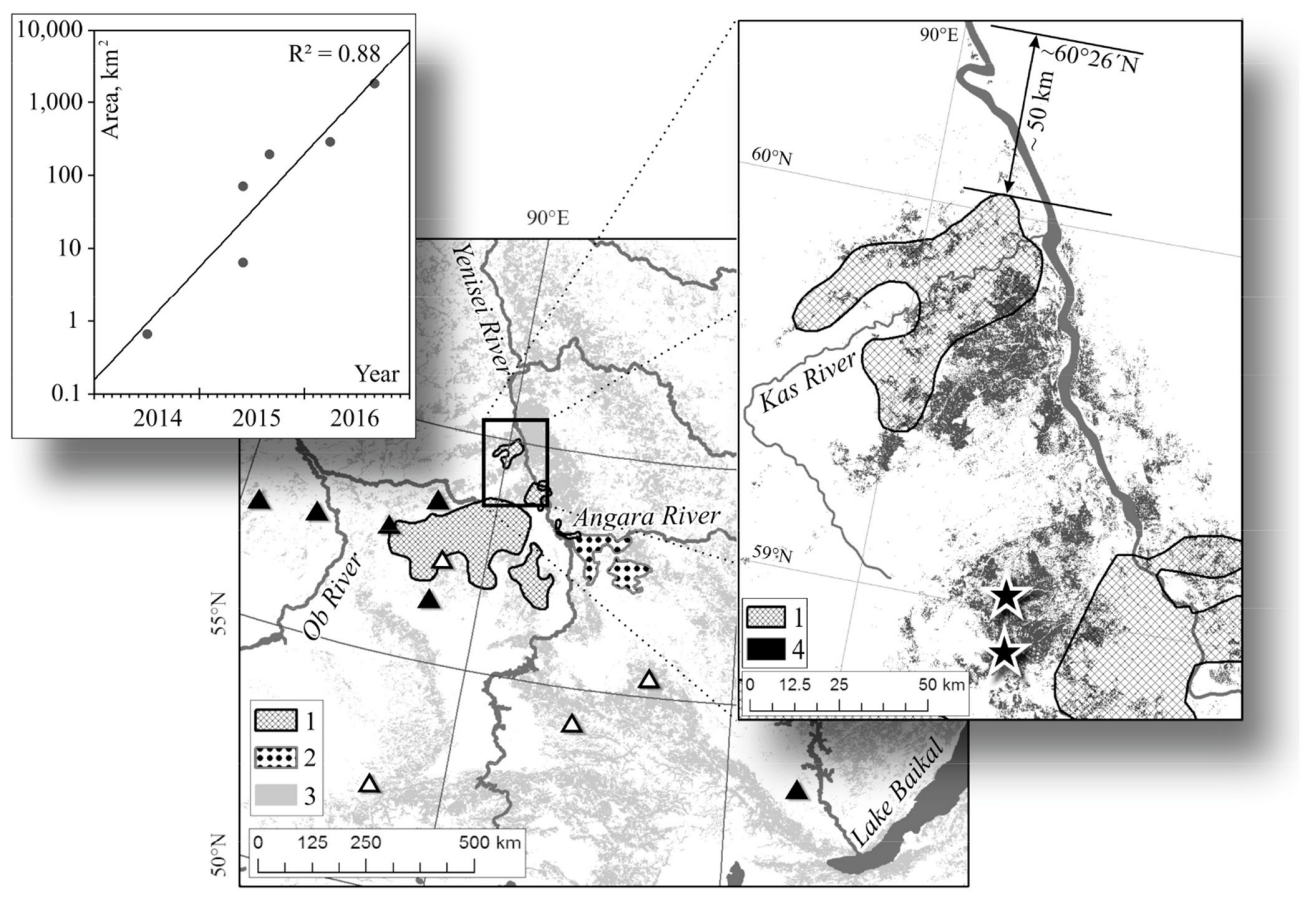
Location (rectangle) of the current (“Yenisei”) Siberian silkmoth outbreak. White triangles: location of earlier observed pest outbreaks; black triangles: outbreaks that occurred in 2014–2015. 1, 2—largest outbreaks: “Ket-Chulym” (1951–1957) and “Priangar’e” (1994–1996), respectively; 3—“dark needle conifer taiga”, composed of *Abies sibirica*, *Pinus sibirica* and *Picea obovata* (according to the map of Bartalev et al. [17]). Inset (right): 1—enlargement showing the northern boundary of “Ket-Chulym” outbreak (former most northward known pest outbreak); 4—dead stands within the “Yenisei” outbreak area. Initial (primary) Yenisei outbreak locations are shown by stars. Arrow indicates the distance between former northern outbreak boundary and northern boundary of the “Yenisei” outbreak. Inset (left): time series of dead stands area dynamics.

Siberian silkmoth (*Dendrolimus sibiricus* Tschetv.) outbreaks were historically observed within the range of 52° N and ≈59° N latitude [1]. The maximal known outbreak area reached 2.5 × 10^6^ ha (1953–57 year, Figure 1) [2]. Preferred food tree species for Siberian silkmoth are fir (*Abies sibirica*) and Siberian pine (*Pinus sibirica*), but the insect also feeds on spruce (*Picea obovata*) and larch (*Larix* sp.). It’s considered that pest outbreaks are induced by droughts, low precipitation and increased air temperatures at the beginning of vegetative period [3]. The northern boundary of outbreaks was approximated by the sum of temperature (*t* > +10 °C) equal to 1200–1400 °C by Rojkov [4]. The Siberian silkmoth outbreak cycle includes a beginning phase, prodromal, eruption (or the outbreak itself), and decreasing phases. Mean eruption and decreasing phases length are about three and two years, respectively. The entire outbreak length is about 10 years when about seven pest generations have occurred, including at least two generations with a one-year generation cycle. In the beginning phase caterpillar density is less than one caterpillar per tree, whereas in the eruptive phase it has been reported to reach 20,000/tree [3,5].

Climate change may affect population dynamics of different pest species, may increase outbreak frequency and may facilitate the shift of range to more northerly latitudes and higher elevations [6–8]. For example, northern expansion of the eastern spruce budworm, *Choristoneura fumiferana* in eastern North America may permit greater defoliation and mortality in extensive northern black spruce forests [9]. Douglas-fir tussock moth is expanding in the southwestern United States and into northern Mexico [10]. Gypsy moth, *Lymantria dispar* is predicted to increase its range in Canada [11]. Droughts and increased aridity are considered to be responsible for the catastrophic outbreaks of *Dendroctonus ponderosae* in North American forests [12]. Periodic droughts stimulated *Dendrolimus pini* outbreaks in Germany forests [13]. Water stress weakened fir (*A. sibirica*) in Central Siberia, which lead to a huge outbreak of bark beetle *Polygraphus proximus* and, as a consequence, widespread fir mortality [14]. Expansion of the northward range of *Dendrolimus sibiricus*, also in Siberia, was reported by Kharuk et al. [15].

Siberian forests exist within a global “hot spot” of increasing air temperature that has persisted over the past several decades [16]. As a result, insect populations may experience noticeable changes. The goal of this paper is to describe an analysis of causes and dynamics of an on-going *Dendrolimus sibiricus* outbreak occurring northward of the known pest species range.

We were seeking to answers the following questions: (i) What is the chronology of Siberian silkmoth outbreak? (ii) What is the relationship between outbreak occurrence and climate variables? (iii) What are the potential changes to the geographical range of Siberian silkmoth outbreaks?

## 2. Materials and Methods

### 2.1. Study Area

The majority of silkmoth outbreak areas occurred within southern part of the Yenisei and Ob Rivers watersheds. Outbreaks occurred within other regions of Central and Eastern Siberia almost simultaneously with the Yenisei outbreak, as shown in Figure 1.

Within the Yenisei outbreak area mean annual precipitation is 540 mm (190 mm in summer); mean January and July temperatures are −21 °C and +19 °C, respectively.

The study forests are moss, grass-moss, and shrub-moss types. Stands composed of fir and Siberian pine (dominant species) with presence of spruce (*Picea obovata*), birch (*Betula pendula*) and aspen (*Populus tremula*); the proportion of the latter three species in canopy was about 10–30%. Siberian pine mean age, height and diameter are 213 years, 21 m and 35 cm, respectively. Fir mean age, height and diameter are 95 years, 17 m and 18 cm, respectively.

### 2.2. Data

Satellite (i.e., Landsat 7 and 8, Sentinel-2A, Worldview) and ground-truth data of the Yenisei outbreak area were analyzed to identify local outbreak areas and assist in the analysis of outbreak site characteristics. Landsat digital data were obtained from the United States Geological Survey (USGS) GloVis web-site (http://glovis.usgs.gov). Sentinel-2A scenes (described by https://scihub.copernicus.eu/dhus) with spatial resolution 10 m (blue, green, red, and NIR) were used. High-res (0.5 m) Worldview scenes were obtained from https://www.bing.com/mapspreview and https://www.google.com/maps and were used primarily as visual references of local site conditions. A total of fifteen Landsat and two Sentinel-2A scenes were analyzed spanning the years 2013–2016.

### 2.3. Image Analysis

ESRI ArcGIS (http://www.esri.com) and Erdas Imagine (http://geospatial.intergraph.com) software were used for image analysis. Images were mosaicked and projected onto UTM (zone 46) reference. A mask of dark needle stands acquired prior to the outbreak (2013) was generated based on expert knowledge and a supervised classification of Landsat and Worldview scenes. The maximum likelihood classification method with a threshold procedure (threshold *p* = 0.05) was used to generate classification map of “dark needle stands” (composed by *A. sibirica*, *P. sibirica* and *P. obovata*). In total, fourteen representative samples of dark needle stands were selected (about 13,700 pixels per sample). The mean Jeffries-Matusita distance between samples was 1040 (confusion error ≈23%). Area of the dark needle stands was 18,250 km^2^ (≈18.5% of the analyzed area). Classification maps of dead and dying stands (with >75% and >50% of dead trees, respectively) were generated for the years 2014–2016.

The SRTMGL1 digital elevation model (horizontal spatial resolution 30 m, vertical 8.6 m; available at (https://lpdaac.usgs.gov/dataset_discovery/measures/measures_products_table/srtmgl1v003) was used to analyze the spatial distribution of stands (i.e., with respect to elevation, exposure and slope steepness). First, topographic normalization was applied to all scenes based on the C-algorithm [18]. Topographic features (elevation, slope steepness, aspect) were analyzed with discretization of 100 m, 5°, and 45°, respectively.

Ground-truth data were collected by specialists from the Forest Defense Center of Krasnoyarsk Region. Data included species composition, canopy closure, dominant species, age, diameter at breast height (dbh = 1.3 m), height, and tree vigor, regeneration and soil type. The climate and ecological variables used in the analysis of pest outbreak occurrence are presented in Table 1.

Drought was analyzed based on SPEI (The Standardized Preripitation-Evapotranspiration Index) data which were obtained from http://sac.csic.es/spei (spatial resolution 0.5° × 0.5°). SPEI is based on this difference (*D_i_*) between precipitation amount (*P_i_*) and potential evapotranspiration (*PET_i_*), where *i*—posiod, data are normalized in space and time [19]:

$$D_{i}=P_{i}-{\mathrm{PET}}_{i}$$

## 3. Results and Discussion

### 3.1. Siberian Silkmoth Outbreak Chronology in Central Siberia

**Figure 2.**
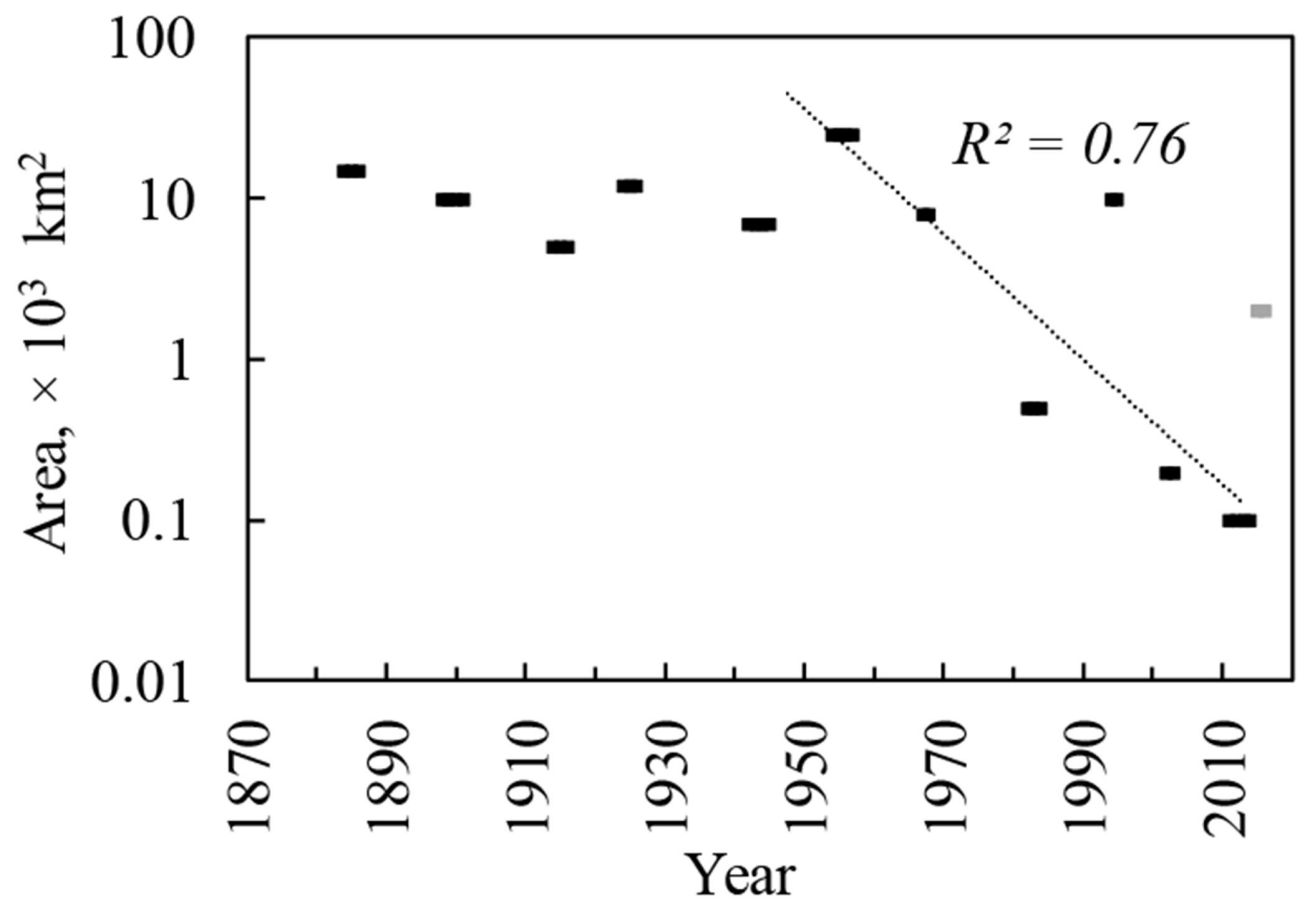
Siberian silkmoth outbreak chronology (according to [2,3,14,20–22]). Trend shown without latest outbreak (which is shown by light gray mark).

Recorded pest outbreaks within Central Siberia have covered an area up to 1.0 × 10^6^–2.5 × 10^6^ ha. Since the catastrophic outbreak in 1951–1957 (2.5 × 10^4^ km^2^), outbreak area has decreased Figure 2). This can be attributed to a reduction in the Siberian silkmoth “food base” caused by earlier outbreaks, as well as a decrease in forest stand size and increased fragmentation due to logging. The “Yenisei” outbreak discussed here has already affected intact stands northward of the historical latitudinal boundary of Siberian silkmoth outbreaks (Figure 1): Within the Yenisei outbreak area silkmoth have reduced generation length from two years to one year, a fact that is not trivial nor obvious for the northern Siberian silkmoth. population.

### 3.2. Outbreak Relevance to Relief Features

**Figure 3.**
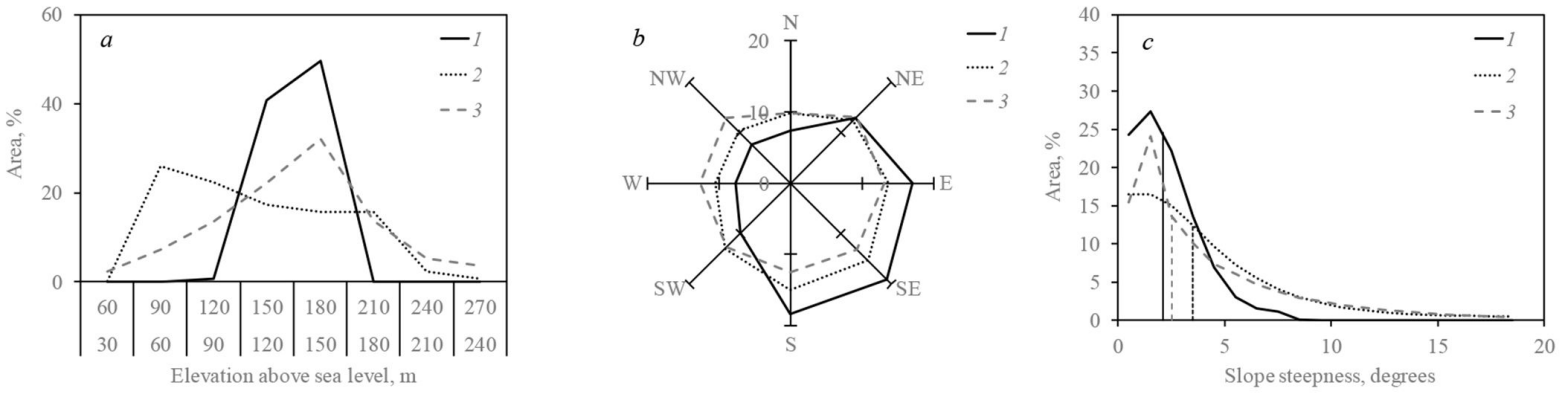
Distribution of dead and of outbreak primary locus stands within outbreak area with respect to (**a**) elevation; (**b**) azimuth; and (**c**) slope steepness (medians shown by vertical lines). 1—outbreak primary locus; 2—all dead stands; 3—all analyzed area. Data normalized with respect to % of all stands.

The initial Yenisei Silkmoth outbreak was discovered in 2014. Later topographic analysis showed that the outbreak site was in an area of gentle (up to 5°) slopes of south-west exposure with elevation about 150–180 m (Figure 3a). Within two years forest damage spread to elevations up to 350 m mostly on slopes of northern exposure with steepness up to 25° (Figure 3b,c). A ten-fold increase in area occurred between July 2014 and July 2015 (≈1–10 km^2^) and another tenfold increase (≈10–100 km^2^) during July of 2015. By the year 2017 total outbreak area reached about 800,000 ha with conifer mortality within about 300 thousand ha with a trend of increasing area of damaged stands.

### 3.3. Pest Outbreak and Climate Variables

**Figure 4.**
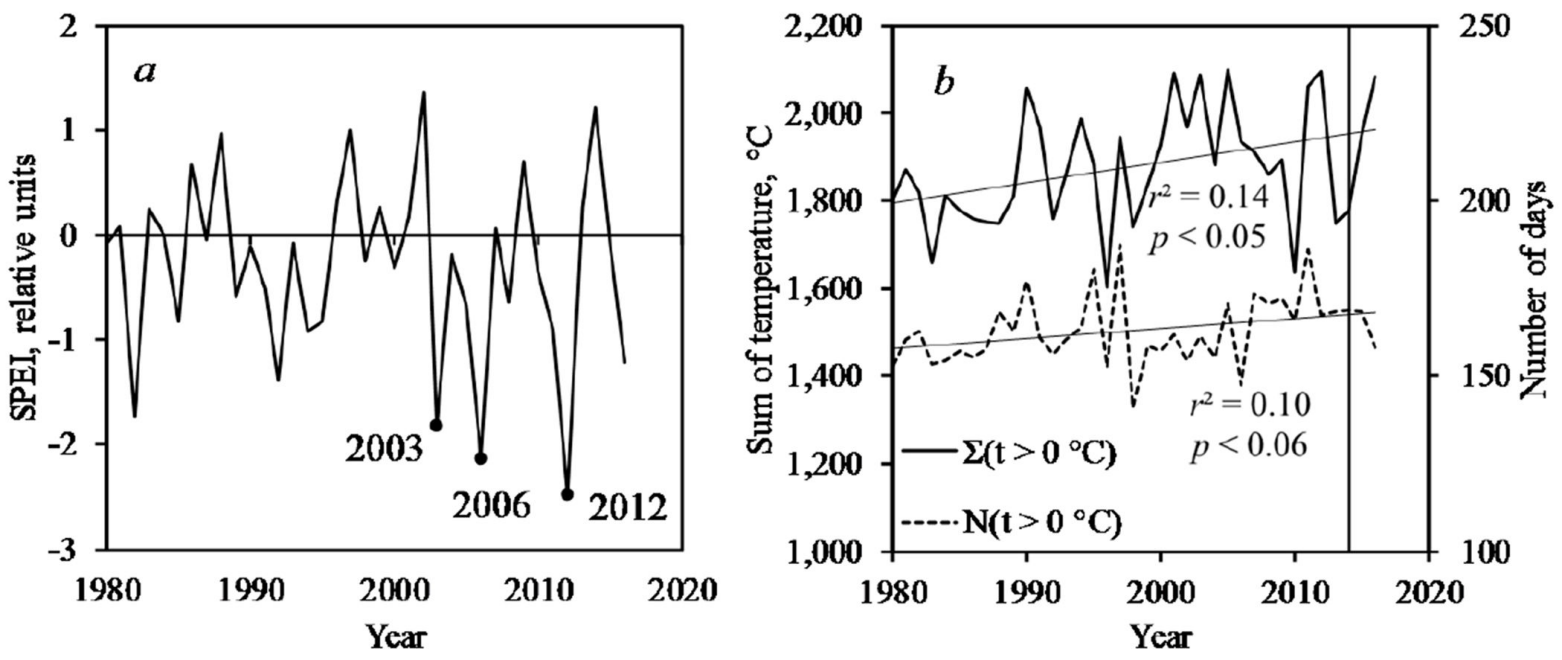
Climate variables dynamics within the zone of Yenisei pest outbreak, (**a**) Standardized Precipitation-Evapotranspiration Index (SPEI, which indicated drought years); (**b**) sum of *t* > 0 ° C and number of days with *t* > 0 °C.

Within the study area, significant changes were observed for drought index SPEI, sum of positive (*t* > 0 °C) temperatures and the number of days with positive temperatures (Figure 4). Since 2000, the latter two variables increased to +120 °C and +9 days, respectively (reference period: 1980–1999) (Figure 4b). Along with that, drought severity and frequency increased (years 2003, 2006, 2012; Figure 4a).

**Figure 5.**
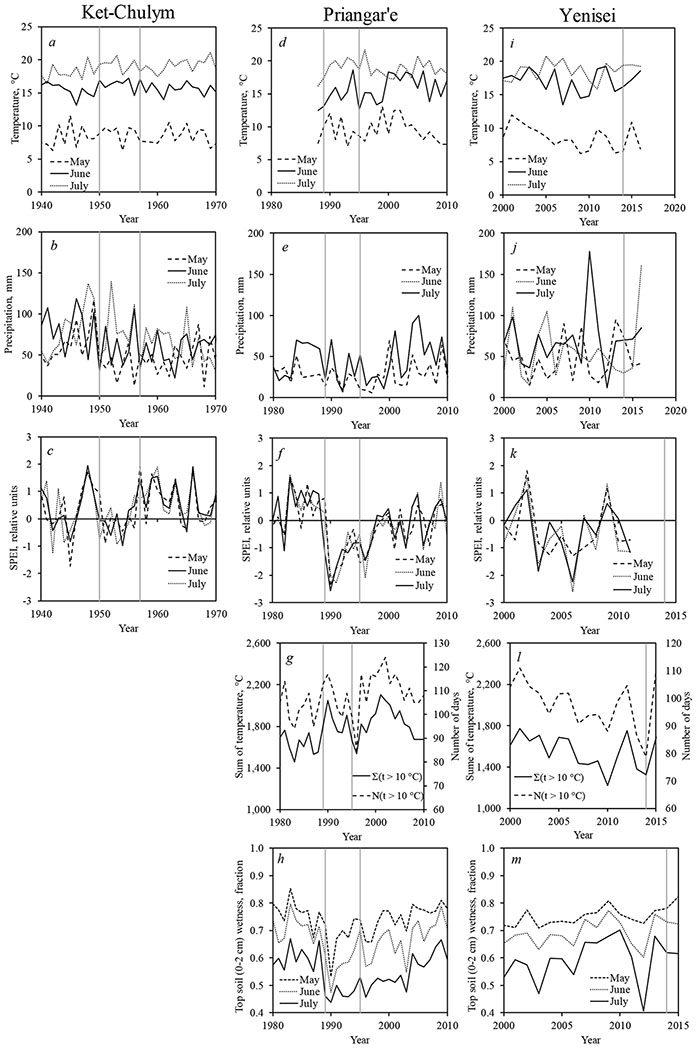
Climate variables dynamics before and during “Ket-Chulym” (**a**–**c**); “Priangar’e” (**d**–**h**) and “Yenisei” (**i**–**m**) pest outbreaks. Vertical lines indicated beginning and end of outbreak. (**a**,**d**,**i**)—temperature; (**b**,**e**,**j**)—precipitation; (**c**,**f**,**k**)—SPEI; (**g**,**l**)—sum of temperature; (**h**,**m**)—top soil wetness.

Climatic time series are presented for areas where earlier major outbreaks occurred (Ket-Chulym and Priangar’e) within the historical outbreak range of the insect, and for the current Yenisei outbreak (Figure 5). Outbreaks were generally preceded by an increase in the sum of positive (Figure 5g,l) and of May–June air temperatures (Figure 5a,d,i), as well as by a decrease in precipitation (Figure 5b,e,j), an increase in the drought index (Figure 5c,f,k), and a decrease in top-soil moisture (Figure 5h,m). Notably, those outbreaks were preceded or coincided with drought years. This pattern was true for all outbreaks that occurred in the period 1950–2015.

### 3.4. Northern Boundary of Siberian Silkmoth Range

**Figure 6.**
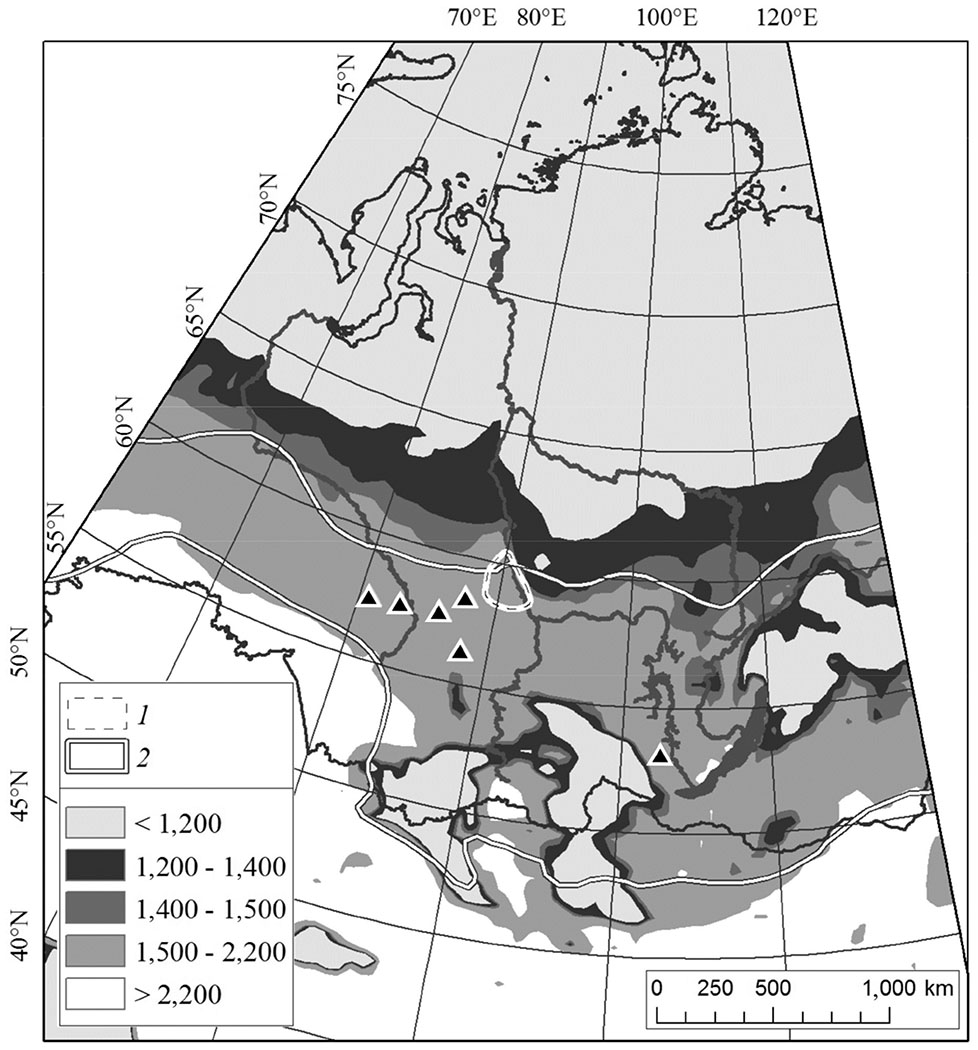
Sum of daily temperatures *t* > 10 °C (Σ_*t*>+*10*°*C*_, 2011–2016 mean). 1—the current Yenisei outbreak (white-dashed line). 2—historical Siberian silkmoth climatic envelope [4], modified by Baranchikov and Kondakov (http://forest.akadem.ru/projects/c2/ (solid white lines). Triangles indicate outbreaks that appeared in 2014–2015.

According to empirical observations [4], the historical northern boundary of the Siberian silkmoth range was approximated by the geographical limit of cumulative temperatures above 10 °C exceeding 1200 °C, whereas the southern boundary was approximated at summation exceeding 2200 °C. The insect climatic envelope is presented in Figure 6, based on the air temperature data of the 1960s. As it can be seen in Figure 6, the 21st century temperature northern limit shifted northward by about 150–500 km. The Yenisei outbreak area can be observed mostly above the historical climatic envelope of the Siberian silkmoth.

The Yenisey outbreak facilitated northward migration of the secondary pests that attacked weakened by Siberian silkmoth stands. Alongside with traditional enemy of “black taiga”, *Monochamus urussovi*, an aggressive *Polygraphus proximus* species was activated in fir-dominant stands in Siberia. That bark beetle was responsible for massive dieback of water stress-weakened fir stands in the southern taiga [23].

Along with northward migration, warming promotes opportunities for Siberian silkmoth migration into mountainous areas, where low temperatures tend to limit insect population. In the beginning of the 21st century, the upper silkmoth boundary was below 550 m above sea level (a.s.l.) [14,22]. Meanwhile warming at higher elevations leads to prolongation of vegetation period, and increase of tree growth increment and stand closure, as well as an upward advance of treeline [24]. Thus, pest outbreaks above the historical boundary should be expected in the nearest future. Notably, insect migration along an elevation gradient was recently reported in US forests [12].

## 4. Conclusions

Climate change promotes migration of Siberian silkmoth outbreak range by about one degree latitude northward of the historical record. Increased warming (ΔΣ_*t*>+*10*°*C*_ = +120 °C), vegetation period length (+9 days) and aridity increase promoted silkmoth invasion into intact northern “black taiga” forest. At present “temperature boundary” (Σ_*t*>+*10*°*C*_ >1200 °C) of potential outbreaks shifted northward at a distance of about 150–500 km in comparison with the boundary defined in the 1960s [4]. The Yenisei silkmoth outbreak considered here covered an area of about 800 thousand ha with conifer mortality within an area of about 300 thousand ha. Similar large-scale outbreaks hardly possible within southward taiga because pest “food base” was exhausted by former outbreaks and anthropogenic forest fragmentation. Within outbreak area silkmoth have reduced generation length from two to one year, the fact that is not trivial for the northern Siberian silkmoth population. The Yenisei outbreak is part of the panzonal (wide-spread) silkmoth outbreak in southern Siberia with distance between outbreaks locations >1000 km along latitude (Figure 6). Finally, the northward migration of silkmoth will facilitate wildfire rate because pest-caused stands mortality strongly increases fire frequency and burned area (up to ten times with comparison with intact stands [25].

## Figures and Tables

**Table 1.** Variables and datasources.

N	Dataset Name	Data Source	Spatial Resolution	Time Coverage	Variables Used
1	Taseevo meteo station	http://meteo.ru	Point	1988–2016	Temperature, precipitation (distance from outbreak ~50 km)
2	Yeniseisk meteo station	http://climexp.knmi.nl	Point	1871–2016	Temperature, precipitation (distance from outbreak ~90 km)
3	CRU TS 3.23	https://crudata.uea.ac.uk/cru/data/hrg/cru_ts_3.23	0.5° × 0.5°	1901–2014	Temperature, precipitation
4	GHCN CAMS	https://www.esrl.noaa.gov/psd/data/gridded/data.ghcncams.html	0.5° × 0.5°	1948–2016	Temperature
5	GPCC	https://www.esrl.noaa.gov/psd/data/gridded/data.gpcc.html	0.5° × 0.5°	2013–2016	Precipitation
6	MERRAM M2SDNXSLV. 5.12.4	ftp://goldsmr4.sci.gsfc.nasa.gov//data/s4pa/MERRA2_MONTHLY	0.5° × 0.625°	1980–2016	Daily temperature Top soil (0–2 cm) moisture
7	SPEI	http://sac.csic.es/spei	0.5° × 0.5°	1950–2016	Dcought indsx
